# Characteristic Muscle Quality Parameters of Male Largemouth Bass (*Micropterus salmoides*) Distinguished from Female and Physiological Variations Revealed by Transcriptome Profiling

**DOI:** 10.3390/biology13121029

**Published:** 2024-12-08

**Authors:** Qingchun Wang, Siqi Lu, Yifan Tao, Jixiang Hua, Yan Zhuge, Wenhua Chen, Jun Qiang

**Affiliations:** 1 Wuxi Fisheries College, Nanjing Agricultural University, Wuxi 214081, China; chun22315@163.com (Q.W.);; 2 Key Laboratory of Freshwater Fishes and Germplasm Resources Utilization, Ministry of Agriculture, Freshwater Fisheries Research Center, Chinese Academy of Fishery Sciences, Wuxi 214081, China; 3 Suzhou Aquatic Technology Extension Station, Suzhou 215004, China

**Keywords:** *Micropterus salmoides*, male and female, amino acid, fatty acid, histological analysis, transcriptomics

## Abstract

Male largemouth bass (*Micropterus salmoides*) are often overlooked because females grow faster. We explored the value of male largemouth bass by comparing muscle nutrition, texture, and transcriptomes between males and females. Females grew faster than males (*p* < 0.05) because of lipid accumulation. Male fish muscles had higher contents of serine, valine, methionine, arginine, nervonic acid, and α-linolenic acid (*p* < 0.05), and female fish muscles had higher contents of aspartic acid, glycine, cysteine, leucine, palmitic acid, docosahexaenoic acid, and 11,14,17-eicosatrienoic acid (*p* < 0.05). Male muscles had a higher concentration of collagen fibers and greater shear force, indicative of a chewier texture. Male muscles had a lighter color, suggesting that they were less susceptible to oxidation and deterioration. Transcriptomic analyses revealed upregulation of *lpl*, *sadb*, *dgat2*, *bhmt*, *tecrb*, and *hsd3b7*, encoding components of amino acid and fatty acid metabolism; and upregulation of *akt2*, *src*, and *kras*, encoding crucial regulators of cellular immunity and homeostasis, in male muscles. Immunity-related pathways, including apoptosis, ErbB signaling, and cellular senescence, were enriched in male fish muscles, indicating heightened immune function. The muscles of male fish have a unique profile and distinctive advantages in terms of nutrition, flavor, texture, and transcriptional regulation.

## Simple Summary

Female largemouth bass grow faster than males; hence, females are often favored for aquaculture. We analyzed the muscle characteristics of male largemouth bass in terms of nutrient contents, texture, and gene transcription. We found that the muscles of male largemouth bass have a unique amino acid and fatty acid profile and contain more collagen than female muscles. Furthermore, pathways related to immunity were enhanced in the muscles of males. We concluded that the muscles of male largemouth bass exhibit distinctive nutritional and textural characteristics and show enhanced disease resistance; thus, they have potential value as a product in their own right.

## 1. Introduction

Largemouth bass (*Micropterus salmoides*), originally native to the freshwater basins of North America, exhibits rapid growth, robust stress tolerance, and broad adaptability. This fish species is extensively farmed in China [1]. The growth rate of female largemouth bass is faster than that of their male counterparts [2], indicative of sexual dimorphism. Sexual dimorphism refers to the phenomenon whereby there are significant differences in economic traits, such as size and growth rate, between males and females of the same organism [3]. Numerous studies have demonstrated the superior growth rate of female largemouth bass compared to males, but there is evidence suggesting that male largemouth bass can exhibit a growth rate that is comparable to, or even greater than, that of females [4]. Thus, although male largemouth bass are traditionally considered to be less valuable than females, the idea that females exhibit superior growth performance is not entirely consistent with the evidence.

Besides growth rate, the nutritional requirements and muscle nutrient levels can differ between male and female fishes because of differences in their reproductive strategies [3]. Female fish tend to allocate more energy to spawning, resulting in higher concentrations of fat and fatty acids in the muscles compared with those in male fish [5]. However, in some instances, the fatty acid content in muscles is higher in males than in females. For example, male red claw crayfish were found to have significantly higher concentrations of delicious amino acids (DAA) in the muscles [3]. The concentrations of DAA, essential amino acids (EAA), and total amino acids (TAA) in the muscles were found to be significantly higher in male Chinese hook snout carp (*Pelodiscus sinensis*) than in female individuals [6]. Variations in muscle nutrient composition can affect its structure. Specifically, the fat and amino acid contents affect muscles’ fiber density and toughness [7,8]. Fat content can also affect muscle quality indicators, such as pH, water-holding capacity, tenderness, and meat color [9,10], suggesting that the muscles of male fish may have a superior flavor than that of female fish. However, little is known about the differences in muscles’ nutrition, structure, and meat quality between male and female largemouth bass.

The differential expression of certain genes induced by sexual factors is considered to be the primary determinant of phenotypic variations between male and female individuals [11]. The differentially expressed genes (DEGs) between sexes can explain differences in growth and muscle nutrition between male and female individuals. Analyses of these genes have shed light on the molecular mechanisms governing differential amino acid and fatty acid accumulation in tissues between males and females. For instance, higher transcript levels of *pomc*, encoding pro-opiomelanocortin, were found to be related to the inhibition of feeding behavior in female tilapia, providing a transcriptomic explanation for their slower growth. Farnesyl diphosphate synthase, encoded by *fdps,* can effectively enhance fat metabolism in the fish body, and the activation of the AMPK signaling pathways can lead to increased amino acid and fatty acid accumulation [12,13,14]. Comparative transcriptome analyses can reveal distinct expression patterns in a specific organ or tissue, thereby elucidating the molecular mechanisms underlying phenotypic differences between male and female populations.

Because female largemouth bass grow faster than males, most research to date has focused on female fish. However, there is insufficient research on the differences in muscles’ nutrition and transcriptomes between male and female largemouth bass. Consequently, it is difficult to adequately assess the value of male and female largemouth bass, and the males are not as highly valued. The aim of this study was to analyze the muscles’ nutrition and texture characteristics of male largemouth bass, to identify any advantages unique to males, and to explore the reasons for these differences by analyzing male and female muscles’ transcriptomes. The results of this study provide a theoretical reference for the development of the single-sex culture of largemouth bass.

## 2. Materials and Methods

### 2.1. Fish and Feeding Management

The experimental fish were selected from the Freshwater Fisheries Research Center of the Chinese Academy of Fishery Sciences (Wuxi, China). We selected largemouth bass at 60 days post-hatching with an average body weight of 56.25 ± 0.18 g, and then the sex of each fish was determined using molecular markers previously developed in our laboratory [15]. The fish were segregated by sex and temporarily housed in tanks (210 cm × 210 cm × 150 cm) in indoor recirculating aquaculture systems with 20 tails in each tank, with each group consisting of four replicates (80 tails per group). Both male and female fishes were cultured in a circulating aquaculture system maintained at 24.0 °C ± 0.3 °C, with ammonia-nitrogen levels below 0.01 mg/L and a dissolved oxygen concentration of >6 mg/L. The female and male fish were provided with a commercial diet containing 46.0% crude protein and 6% crude fat twice daily, at 8:00 a.m. and 16:00 p.m., in quantities equivalent to 2% of body weight. The feeding cycle lasted for 135 days.

### 2.2. Sample Collection

After a 24-h fasting period, the fish were anesthetized with 200 mg/L MS-222 prior to sampling. Twenty individuals (five per replicate) were randomly collected from both male and female populations. The weight of each fish was measured using an electronic scale with an accuracy of 0.01 g. The weight gain rate and feed efficiency were calculated as follows:

weight gain rate%=final weight−initial weightinitial weight∗100%

feed efficiency=final weight−initial weightfeeding amount∗100%

After removing the scales and skin tissue, the muscles on both sides of the fish body were cut across at the base of the dorsal fin, with the spine serving as the boundary. The surface moisture was then absorbed using filter paper, and the dissected muscle was weighed to obtain net meat weight. Finally, the fillet yield was calculated as follows:

fillet yield=net meat weightbody weight∗100%

Immediately after weighing, the muscle tissue (2 cm from the head) was cut from the same location on the same side of each specimen using a scalpel. Muscle tissue samples were collected from six randomly selected fish per group and fixed in 4% *v*/*v* paraformaldehyde for histological analysis. Another six muscle samples (0.5 cm × 0.5 cm × 1 cm) per group were collected for immediate analysis of fillet quality. An additional eight samples per group were randomly sectioned and immediately frozen in liquid nitrogen before being stored at −80 °C. Of those eight samples, four were used for nutrient analyses, and the other four were separated into two parts for RNA extraction for transcriptome sequencing and for gene expression verification.

### 2.3. Proximate Composition of Muscle Samples

Moisture, crude protein, crude lipid, and ash contents in muscles were analyzed following the methods recommended by the Association of Official Analytical Chemists (AOAC, 2006) [16]. The moisture content was determined using the drying method at 101.3 kPa and 105 °C, while the crude protein content (6.25 N-to-protein conversion factor) was determined using the Kjeldahl method. The crude lipid content was determined using the Soxhlet extraction method. To determine the ash content, each sample was first burned in an electric furnace and then in a muffle furnace (550 °C, 6 h).

### 2.4. Determination of Amino Acid Composition

The TAA profiles in muscle samples were determined by the method described by Wu et al. [17]. Each freeze-dried muscle sample (30 mg) was added to a 6 mol/L hydrochloric acid solution, frozen under vacuum, and then hydrolyzed at 110 °C for 22 h. After hydrolysis, the sample was diluted with distilled water to a final volume of 50 mL. One milliliter of the diluent was accurately absorbed and dried at 50 °C before being rehydrated with 2 mL of water and dried again under pressure. After drying, the residue was dissolved in a 2 mL buffer solution containing sodium citrate with a pH of 2.2. The resulting solution was filtered through a 0.22 μm membrane and then analyzed using an automatic amino acid analyzer (LA8080, Hitachi, Tokyo, Japan) to determine the concentration of amino acids in the sample. The concentration of each amino acid was determined from the peak area by comparison with an external standard.

### 2.5. Determination of Fatty Acid Composition

The fatty acid composition was determined, as described by Johnston et al. [5], with some modifications. Total muscle lipids were extracted by hydrolysis. Specifically, each 1.2 g freeze-dried sample was added to an 8.3 mol/L hydrochloric acid solution and then hydrolyzed at 70 °C for 40 min. The resulting mixture was neutralized with 95% ethanol and extracted using a combination of ethyl ether and petroleum ether. Total lipids were obtained through concentration to dryness using a rotary evaporator (N-1300S, EYELA, Tokyo Rikakikai Co., Ltd., Tokyo, Japan). The total lipids were subjected to methylation to generate fatty acid methyl esters (FAMEs) using a 2% sodium hydroxide and formaldehyde (*w*/*v*) solution. The internal standard used during the methylation process was C19:0. The FAMEs were isolated and characterized using capillary chromatography (column length 100 m, inner diameter 0.25 mm, film thickness 0.2 μm) on a gas chromatograph (7890A, Agilent, Santa Clara, CA, USA). The gas chromatography procedure was as follows: initial temperature of 100 °C for 13 min, followed by heating at 10 °C/min from 100 °C to 180 °C, maintained at 180 °C for 6 min; heating at 1 °C/min from 180 °C to 200 °C, maintained at 200 °C for 20 min, heating at 4 °C/min from 200 °C to 230 °C, maintained at 230 °C for 10.5 min. The inlet and detector temperatures were set to 250 °C, and high-purity helium was used as the carrier gas. The sample was injected at a shunt ratio of 100:1, and the fatty acid content was determined using the peak area percentage method.

### 2.6. Histological Analyses of Muscle Samples

After fixation in 4% *v*/*v* paraformaldehyde for 24 h, the muscle tissue was embedded in paraffin wax, then dewaxed and stained with hematoxylin-eosin (HE) before being dehydrated. The resulting samples were observed under a light microscope, and the size of various components of the muscle tissue was measured using Image-Pro Plus 6.0 software [18].

### 2.7. Measurement of Flesh Quality Parameters

#### 2.7.1. Shear Force

After slicing the muscle sample to a thickness of 10 mm, a texture analyzer (SD-700, Akiyama Technology Co., Ltd., Dongguan, China) was utilized to vertically section the sample. A constant speed of 1 mm/s was maintained during the pre-test and test, and the trigger force in the post-test phase was 5.0 g. Parallel detection was performed at a shear depth of 3 mm for a total of eight times. The maximum force required to cut the samples was recorded as the shear force [10].

#### 2.7.2. Centrifugal Weight Loss

The centrifugal weight loss (%) was used to determine the water-holding capacity of the muscle samples. Each fish muscle sample was weighed (*w*1), wrapped in filter paper, placed in a 7-mL centrifuge tube, and then centrifuged at 4 °C and 10,000× *g* for 15 min (Rotina 420 R, Hettich Lab Technology, Tuttlingen, Germany). Then, the sample was re-weighed (*w*2), and the centrifugal weight loss (%) was calculated as follows:

$$Centrifugal\;weight\;loss\;\left(\%\right)=\left(1-w2/w1\right)*100\%$$

#### 2.7.3. pH

The pH of muscle samples was determined according to Manor et al. [10] with minor modifications. Each muscle sample (4.0 g) was homogenized with 25 mL of deionized water using an ultraturrax (IKA, Staufen, Germany). Following centrifugation at 10,000 r/min for 10 min at 4 °C, the pH of the supernatant was determined using a pH Meter (PHSJ-3F, Leici, Shanghai, China). The pH value was recorded as the muscle pH.

#### 2.7.4. Colour Analysis

Muscle color was measured using a CR400 Chroma Meter (Minolta, Osaka, Japan). Color measurement was conducted using the three-point test method [9]. The results are reported as L* (brightness), a* (+a for red; −a for green), and b* (+b for yellow; −b for blue) values. The W* (whiteness) is calculated using the Hunter whiteness formula as follows:

$$W^{*}=100-{\left\{{\left(100-L^{*}\right)}^{2}+K\left[{\left(a^{*}-a_{p}\right)}^{2}+{\left(b^{*}-b_{p}\right)}^{2}\right]\right\}}^{1/2}$$

where *a_p_* and *b_p_* have a value of 0, and *K* is set to 1.

### 2.8. Sequencing and Analysis of Muscle Transcriptome

#### 2.8.1. RNA Extraction, Isolation and Purification

Total RNA was extracted and purified from four samples of males and females using TRIzol reagent (Invitrogen, Carlsbad, CA, USA). The quantity and purity of total RNA were assessed using a NanoDrop ND-1000 instrument (NanoDrop, Wilmington, DE, USA), and RNA integrity was evaluated using an Agilent 2100 instrument (Agilent, Santa Clara, CA, USA). Only high-quality RNA with a RIN value > 7.0 was used for library construction.

#### 2.8.2. cDNA Library Construction and Sequencing

The Ribo-Zero™ rRNA Removal Kit (Illumina, San Diego, CA, USA) was used to eliminate ribosomal RNA and obtain purified high-quality RNA. The remaining RNA was fragmented at 94 °C for 5–7 min using the Magnesium RNA Fragmentation Module [New England Biolabs (NEB), cat. e6150, Ipswich, MA, USA], and then cDNA was synthesized using reverse transcriptase (Invitrogen). The next step involved the synthesis of U-labeled second-stranded DNAs using *Escherichia coli* DNA polymerase I (NEB), RNase H (NEB), and dUTP solution (Thermo Fisher Scientific, Waltham, MA, USA). An A base was added to DNA ends to form flat ends. The resulting DNA fragments were screened and purified using magnetic beads. After digestion with UDG (NEB), a library of 300 ± 50 bp fragments was generated by PCR. Finally, the Illumina Novaseq™ 6000 (LC-Bio, Hangzhou, China) was utilized to sequence paired-end reads (2 × 150 bp; PE150).

#### 2.8.3. Data Analysis and Bioinformatics Analysis

High-quality clean reads were obtained by filtering raw sequencing data using Cutadapt [19], followed by alignment of clean reads to the largemouth bass reference genome using the HISAT2 package (https://daehwankimlab.github.io/hisat2/, accessed on 25 December 2022, version: hisat2-2.2.1). The mapped reads of each sample were assembled using StringTie (http://ccb.jhu.edu/software/stringtie/, accessed on 25 December 2022, version: stringtie-2.1.6), and the comprehensive transcriptome was reconstructed by merging all transcriptomes from all samples with gffcompare software (http://ccb.jhu.edu/software/stringtie/gffcompare.shtml, accessed on 25 December 2022, version: gffcompare-0.9.8). Differentially expressed genes were identified based on a false discovery rate (FDR) of <0.05 and an absolute fold change of ≥1. Gene Set Enrichment Analysis (GSEA, v4.1.0) and MsigDB were utilized to determine whether specific sets of genes in Gene Ontology (GO) terms and Kyoto Encyclopedia of Genes and Genomes (KEGG) pathways exhibited significant differences between male and female fishes. The GO terms and KEGG pathways with |NES| > 1, NOM *p*-value < 0.05, and FDR *p*-value < 0.05 were considered to be differentially enriched between male and female fishes.

### 2.9. Real-Time Fluorescence Quantitative PCR

Total RNA was extracted from muscle samples using an RNAiso Plus kit (TaKaRa, Dalian, China), and cDNA was synthesized using Prime Script template RT Master Mix (TaKaRa). The internal reference gene was *β*-actin of *M. salmoides*. The primers used to amplify target genes by qRT-PCR are shown in Table S1. The fluorescence quantitative PCR system was configured in accordance with the instructions of the SYBR^®^ Premix Ex Taq assay kit (TaKaRa), and the thermal cycling conditions were as follows: predenaturation at 95 °C for 30 s, followed by denaturation at 95 °C for 5 s; annealing at 60 °C for 30 s; and extension at 72 °C for 30 s (40 cycles). Relative gene transcript levels were calculated using the ΔΔCt method [20], and each reaction was replicated three times.

### 2.10. Data Processing and Analysis

The data were analyzed and processed using SPSS 26.0 (SPSS Inc., Chicago, IL, USA). The normality and homogeneity of the variance of the data were assessed by Shapiro–Wilk’s and Levene’s tests. Student’s *t*-tests were used to determine the significance of differences between males and females, with *p* < 0.05 considered significant. The results are presented as mean ± standard error (mean ± SE). GraphPad Prism 8.0.1(244) software was used to create bar and line charts. All other figures and charts were constructed in R 4.0.4 (https://www.r-project.org/).

## 3. Results

### 3.1. Growth Parameters and Proximate Composition

As shown in Table 1, the weight gain rate and fillet yield were significantly higher in female fish than in male fish (*p* < 0.05). However, there was no statistically significant difference in feed efficiency between male and female fishes (*p* > 0.05). Analyses of muscle proximate composition revealed a significantly higher lipid content in female fish than in male fish (*p* < 0.05). However, the moisture, ash, and crude protein contents in muscles did not differ significantly between male and female fishes (*p* > 0.05).

### 3.2. Amino Acid and Fatty Acid Composition

A total of 17 amino acids were identified (Table 2), including seven EAA and four DAA. Of the EAA in the muscles, valine (Val) and leucine (Leu) were present at significantly higher concentrations in females than in males (*p* < 0.05), while the concentration of methionine (Met) was significantly higher in males than in females (*p* < 0.05). The total EAA content was significantly higher in females than in males (*p* < 0.05). Additionally, the concentrations of aspartate (Asp) and tyrosine (Tyr) were significantly higher in females than in males (*p* < 0.05), while those of glycine (Gly), serine (Ser), and arginine (Arg) were significantly higher in males than in females (*p* < 0.05). There were no significant differences (*p* > 0.05) in the concentrations of non-essential amino acids (NEAA), DAA, and TAA between male and female largemouth bass.

A total of 16 fatty acids were identified in the muscles of male and female largemouth bass (Table 3). However, only five fatty acids exhibited significant differences in their contents between males and females. The concentrations of C16:0, C20:3n6, and C20:3n3 in the muscles were significantly higher in females than in males (*p* < 0.05), whereas the concentrations of C24:1 and C18:3n3 were significantly higher in males than in females (*p* < 0.05). There were no significant differences (*p* > 0.05) in the concentrations of saturated fatty acids (SFAs), monounsaturated fatty acids (MUFAs), and polyunsaturated fatty acids (PUFAs) between males and females.

### 3.3. Histology Analysis

**Figure 1:**
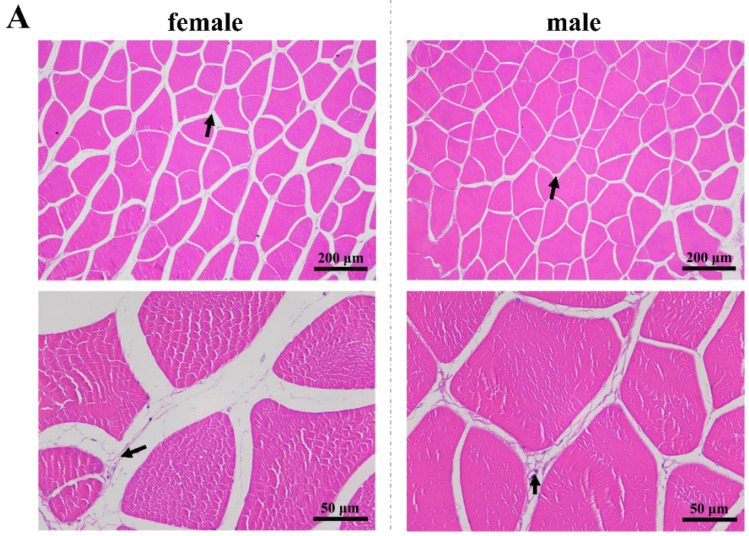

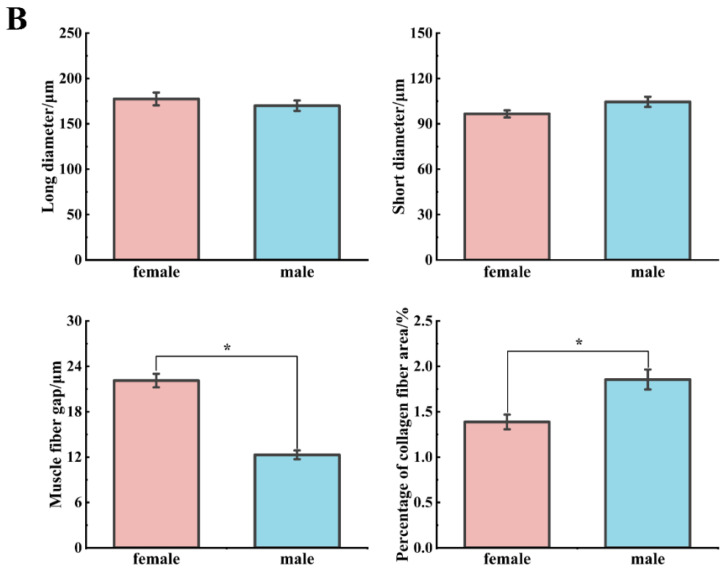
Muscle structures of male and female *Micropterus salmoides*. (**A**) Histopathological sections of female and male muscles at 100× magnification and 400× magnification. (**B**) Analysis of structural differences in muscles between male and female fishes, including long diameter, short diameter, muscle fiber gap, and percentage of collagen fiber area (*n* = 30, 10 myofibers randomly selected from each of three sections in each group). The black arrow indicates the collagen fibers. The asterisk indicates a significant difference (*, *p* < 0.01).

The muscle fibers differed between male and female largemouth bass (Figure 1A). As shown in Figure 1B, there was no significant difference in the long and short diameters of muscle fibers between male and female largemouth bass (*p* > 0.05). However, the muscle fiber gap was significantly larger in females than in males (*p* < 0.05), indicating that the muscle fiber density was lower in females than in males. The collagen fibers were arranged interstitially within muscle fibers, and the male fish exhibited a significantly higher content of collagen fibers compared with their female counterparts (*p* < 0.05).

### 3.4. Meat Quality Parameters

The differences in muscle meat quality between male and female largemouth bass are shown in Table 4. The results of the meat quality analyses indicated that the muscle shear force was significantly higher in males than in females (*p* < 0.05). However, there was no statistically significant difference in centrifugal weight loss or muscles’ pH between males and females (*p* > 0.05). The muscle luminosity L* value differed significantly between males and females (*p* < 0.05) and was 21.33% higher in females than in males. The muscle redness a* value was negative (a* < 0) in females, indicating greenness, but positive (a* > 0) in males, indicating redness, and the difference in muscle a* values between males and females was significant (*p* < 0.05). The muscle yellowness b* value was significantly higher in females than in males (b* < 0, *p* < 0.01).

### 3.5. Transcriptional Analysis

**Figure 2:**
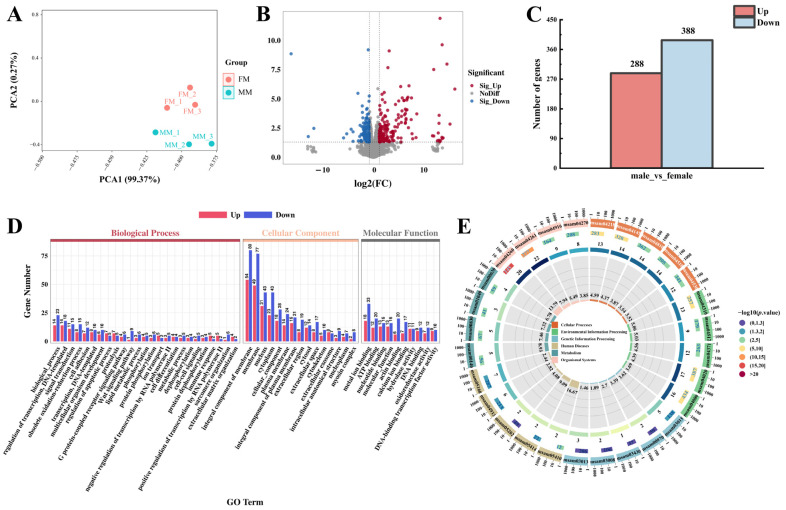
Muscle transcriptome analysis of male and female *Micropterus salmoides* (male vs. female). (**A**) PCA showing significant separation (*p* < 0.05) between male (MM) and female (FM) samples. (**B**) Volcano plot showing DEGs in the muscles of male and female fishes. (**C**) Histogram showing the number of DEGs in muscles between male and female fishes. (**D**) GO annotation analysis of DEGs in muscles between male and female fishes. (**E**) KEGG enrichment analysis of DEGs in muscles between male and female fishes.

Libraries constructed from total RNA extracted from muscle samples of four female fish and four male fish were sequenced on the Illumina platform. This yielded a total of 42,319,828 to 42,697,362 raw reads for female muscle samples and 42,306,324 to 43,221,454 raw reads for the male muscle samples. After filtering, 40,774,216 to 41,295,356 clean reads were obtained for females, and 40,125,512 to 42,065,524 clean reads were obtained for males. The data met the necessary quality standards, with Q20% between 99.97% and 99.98% and Q30% between 96.48% and 96.95% (Table S2). The principal component analysis (PCA) revealed a significant separation between the male and female groups along PCA2 (*p* < 0.05), resulting in the clustering of samples into two distinct groups (Figure 2A). This confirmed that the data were satisfactory for further analyses, such as detection of DEGs, GO annotation, and KEGG enrichment analyses. According to the statistical analysis, there were 676 DEGs in muscles between male and female largemouth bass. Specifically, compared with females, males had 288 upregulated genes and 388 downregulated genes within their muscle tissue (Figure 2B,C).

The DEGs were mapped to the GO database. As shown in Figure 2D, the 676 DEGs were assigned to subcategories within the Biological Process, Cellular Component, and Molecular Function categories. The majority of DEGs were enriched in the Cellular Component category, with 134 DEGs specifically enriched in the “Integral component of membrane” subcategory and another 126 DEGs enriched in the “Membrane” subcategory. The KEGG analysis results revealed significant enrichment of DEGs in multiple pathways (*p* < 0.05). The top 28 enriched pathways are shown in Figure 2E and include “Cardiac muscle contraction” (msam04260), “Cellular senescence” (msam04218), “Tight junction” (msam04530), “Steroid hormone biosynthesis” (msam00140), “ErbB signaling” (msam04012), and other crucial metabolic pathways involved in nutrient metabolism. Additionally, we detected significant enrichment of DEGs in pathways related to amino acid and fatty acid regulation (*p* < 0.05), such as “Biosynthesis of unsaturated fatty acids” (msam01040), “Glycerolipid metabolism” (msam00561), “Tyrosine metabolism” (msam00350), “Glycine, serine and threonine metabolism” (msam00260), and others (Table S3).

### 3.6. Enrichment Analysis of Upregulated DEGs in Muscles of Male and Female

**Figure 3:**
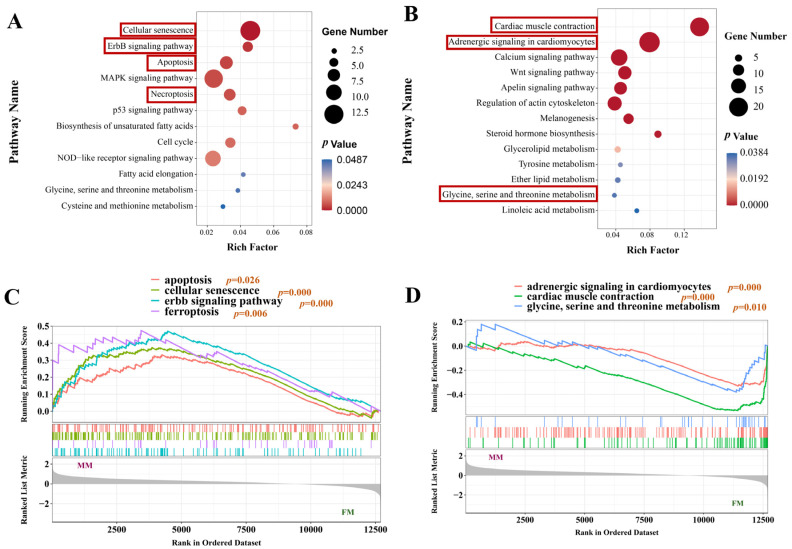
Analysis of upregulated pathways in muscles of male and female largemouth bass. (**A**,**B**) KEGG enrichment analysis of genes showing significant upregulation in the muscles of male (**A**) and female (**B**) fish. (**C**,**D**) GSEA analysis of pathway significant upregulation in the muscles of male (**C**) and female (**D**) fish.

The KEGG enrichment analysis revealed that the upregulated DEGs in female fish muscles were predominantly enriched in pathways associated with muscle contraction and muscle structure (Figure 3A,B). These included the “Cardiac muscle contraction”, “Calcium signaling”, and “Apelin signaling” pathways. The significantly upregulated DEGs in male fish muscles were primarily enriched in the pathways involved in the regulation of cell growth and apoptosis, including the “Cellular senescence” and “Cell cycle” pathways. Additionally, several DEGs were enriched in lipid and amino acid metabolism in the muscles of both male and female fishes, including “Glycerolipid metabolism”, “Tyrosine metabolism”, “Biosynthesis of unsaturated fatty acids”, “Glycine, serine and threonine metabolism”, and other pathways.

The GSEA analysis revealed a significant upregulation of pathways associated with cell proliferation and apoptosis in male fish muscles (*p* < 0.05, Figure 3C), including “Apoptosis”, “Cellular senescence”, “ErbB signaling”, and “Ferroptosis”. Conversely, pathways involved in the regulation of muscle contraction and amino acid metabolism were significantly upregulated in female muscles (*p* < 0.05, Figure 3D), including “Adrenergic signaling in cardiomyocytes”, “Cardiac muscle contraction”, and “Glycine, serine, and threonine metabolism”.

### 3.7. Identification of DEGs Related to Amino Acid and Fatty Acid Metabolism

**Figure 4:**
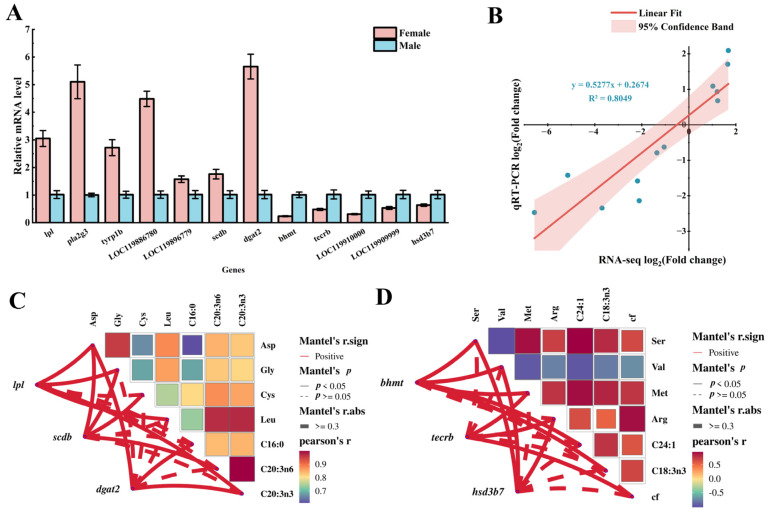
Validation and correlation analysis of key regulatory genes associated with muscle nutrition. (**A**) qRT-PCR verification of gene transcript profiles in the muscles of male and female fishes. (**B**) Correlation analysis of qRT-PCR and RNA-seq based on log2 fold change value. (**C**,**D**) Correlation analysis between upregulated genes involved in amino acid and fatty acid metabolism regulation and muscle content of amino acids and fatty acids in female (**C**,**D**) male fish. cf: collagen fiber. Lines indicate correlations between genes and both amino acids and fatty acids. The network heat map illustrates the inter-relationships between amino acids and fatty acids.

On the basis of the transcriptome sequencing results, DEGs enriched in pathways related to amino acid and fatty acid metabolism in muscles between male and female largemouth bass were selected for validation by qRT-PCR. The results revealed significant upregulation of seven genes (*lpl*, *pla2g3*, *tyrp1b*, *LOC119886780*, *LOC119896779*, *scdb*, *dgat2*) in females compared with males (*p* < 0.01), and significant upregulation of five genes (*bhmt*, *tecrb*, *LOC119910000*, *LOC119909999*, *hsd3b7*) in males compared with females (*p* < 0.01, Figure 4A). The qRT-PCR data were strongly correlated with the RNA-seq data (R^2^ = 0.8049, Figure 4B).

The key DEGs associated with muscle amino acid and fatty acid metabolism in both female and male fish were identified on the basis of gene expression levels, and log2 fold change values. Then, the relationship between DEGs and amino acid fatty acid content was analyzed (Table S4). The results demonstrated a significant positive correlation between *lpl*, *scdb*, and *dgat2* transcript levels and the contents of Asp, Gly, Cys, Leu, and C16:0, C20:3n6, and C20:3n3 in the muscles of female fish (*p* < 0.05, Figure 4C); and a significant positive correlation between *bhmt*, *tecrb*, and *hsd3b7* transcript levels and the contents of Ser, Val, Met, and Arg, and C24:1 and C18:3n3, as well as collagen fibers, in the muscles of male fish (*p* < 0.05, Figure 4D). We also detected some significant relationships between particular amino acids and fatty acids. Specifically, in the female fish muscles, Leu was positively correlated with C20:3n6 and C20:3n3, and in the male fish muscles, Met was positively correlated with C24:1 and C18:3n3.

### 3.8. Identification of DEGs Involved in Different Physiological Functions

**Figure 5:**
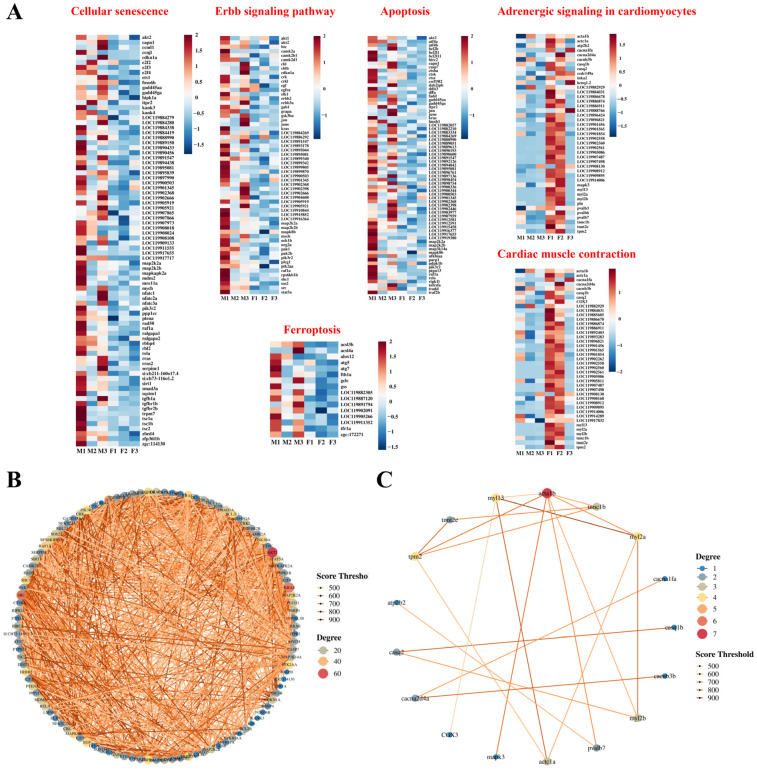
Dynamic expression patterns and hub gene identification in the upregulated pathways identified through GSEA analysis. (**A**) Expression of genes in the leading-edge subset of each gene set. (**B**,**C**) Protein–protein interaction (PPI) network of DEGs and identified hub genes in the muscles of male and female largemouth bass.

The heat map results revealed that genes involved in the regulation of cell proliferation and apoptosis exhibited higher transcript levels in male muscles, whereas genes associated with the regulation of muscle contraction displayed higher transcript levels in female muscles (Figure 5A). To examine gene inter-relationships and identify key genes involved in functional regulation, a protein–protein interaction (PPI) analysis was employed to screen for hub genes (Figure 5B,C). The results demonstrated that the proteins encoded by *akt2*, *src*, and *kras* exhibited the most robust interaction with proteins encoded by other genes. Three genes, namely *akt2*, *src*, and *kras*, were identified as hub genes in gene sets of cell proliferation and apoptosis. Additionally, four hub genes (*acta1b*, *myl13*, *myl2a*, *tpm2*) were identified for muscle contraction.

## 4. Discussion

### 4.1. Differences in Growth and Muscle Nutrient Characteristics Between Males and Females

Differences in reproductive strategies, hormone levels, and survival styles between male and female individuals are the primary factors contributing to disparities in growth rate and meat quality between sexes [3,21]. In this experiment, the weight gain rate and fillet yield of largemouth bass were significantly lower in male than in female fish. However, based on the data analysis, the weight gain rate of male fish was only approximately 13% lower than that of female fish, a difference significantly smaller than those reported in other studies; for example, 100% in yellow catfish (*Pelteobagrus fulvidraco*) and 40% in tilapia (*Oreochromis mossambicus*) [13,22]. Hagen et al. reported that the greater number and density of muscle fibers per unit cross-sectional area in female fish accounted for their significantly higher growth and weight gain rates compared with those of male fish [23]. In contrast, we found that the muscle fiber density was lower in female fish than in male fish because of the wider gap between muscle fibers in female fish. Instead, the significantly higher weight gain rate and fillet yield of female fish may be attributed to their higher lipid content. Studies indicate that female fish tend to accumulate more fat to meet reproductive demands [5], resulting in lipid accumulation within the muscles in female fish. The lipid content in the muscles was 24% higher in female largemouth bass than in male ones in this study, which could account for the higher weight gain rate and meat yield of the females.

The value of meat is determined by its nutritional composition and texture, and the nutrient value is usually assessed based on amino acid and fatty acid contents. The EAA serves as a crucial indicator of protein quality [17]. In this study, the EAA content in muscles was significantly higher in females than in males. However, certain EAA were present at higher levels in males or females; specifically, the Val and Leu contents were higher in female muscles, and the Met content was higher in male muscles. Thus, the muscles of male and female largemouth bass have unique amino acid profiles. The DAA, including Asp, Glu, Gly, and Ala, contribute to the umami (mainly determined by Asp and Glu) and sweet (mainly determined by Gly and Ala) flavors of proteins [24]. Our results indicate that the muscles of female fish likely have a stronger umami flavor (due to Asp), and those of male fish are likely to be sweeter (due to Gly).

The composition of fatty acids is an important factor in the nutritional evaluation of dietary fat sources [25]. Many studies have demonstrated differences in the fatty acid composition of muscles between female and male fish [3,5,6]. In this study, however, only a few fatty acids in muscles exhibited significant differences between male and female fishes. Females had higher levels of C16:0, C20:3n6, and C20:3n3 in the muscles, while males had higher levels of C24:1 and C18:3n3. There were no significant differences in the levels of SFA, MUFA, and PUFA in muscles between male and female fishes. This may be attributed to the feeding and management practices used in this study. The feeding level affects the relative concentrations of saturated fatty acids within fish muscle tissue. In this study, both male and female largemouth bass had a feed coefficient of >1.2, and all fish consumed one type of feed. This may account for the lack of significant differences in muscle fatty acid contents between male and female fishes.

Gene transcript levels are a significant contributing factor to variations in muscle nutrient composition [26]. The KEGG analysis results revealed the enrichment of regulatory pathways associated with amino acid metabolism in male fish, as well as pathways related to fatty acid metabolism in the muscles of female fish. The enrichment of these pathways may contribute to the distinctive amino acid and fatty acid profiles observed in male and female largemouth bass [27]. The level of C18:2n6, an essential fatty acid for human nutrition [28], was higher than that of other fatty acids in both sexes of largemouth bass. These findings are consistent with the enrichment of the linoleic acid metabolism pathway in the muscles of female fish. Some pathways related to unsaturated fatty acid biosynthesis and fatty acid elongation were enriched in the muscles of male fish, which may have contributed to the elevated levels of C18:3n3, C20:3n3, and C20:3n6. The enrichment of lipid metabolic pathways, including glycerolipid metabolism and ether lipid metabolism, can significantly enhance anabolic processes related to the synthesis of steroids, triglycerides, ether lipids, and other lipids within female muscle tissue [29,30]. This may have contributed to the higher lipid levels in the muscles of female fish in this study.

The genes identified within the aforementioned pathways were subjected to correlation analysis. A positive association was detected between the upregulation of these genes and increased levels of amino acids and fatty acids. The upregulation of *scdb* and *lpl* has been demonstrated to enhance the rate of PUFA synthesis [31,32], and increased expression of *bhmt* has been demonstrated to enhance the functionality of the remethylation pathway, thereby augmenting cellular Met content [33]. Those findings align with the results of our study. Our findings indicate that certain genes may participate in the biosynthesis of both amino acids and fatty acids, although the underlying mechanism remains unclear. Both *lpl* and *dgat2* play crucial regulatory roles in lipid synthesis [34,35]. Another study demonstrated that *lpl* overexpression leads to a substantial enhancement in lipid content in the muscles of tilapia (*Oreochromis niloticus*) [36]. The upregulation of genes involved in the regulation of lipid metabolism in the muscles of female fish may have enhanced the rate of lipid synthesis, resulting in significantly higher lipid content compared with male fish. These findings indicate that, although male largemouth bass exhibited slightly inferior growth performance compared with that of their female counterparts, the muscles of both sexes have distinct amino acid and fatty acid profiles and are capable of fulfilling the nutritional requirements of diverse populations.

### 4.2. Flesh Quality Characteristics of Male Differ from Females

Muscle fibers are separated by collagen and adipose tissue. The structure of muscle fibers is intimately linked to both elasticity and chewiness as characteristics of food texture [37]. Larger amounts of intermuscular connective tissue and lipids result in larger gaps between muscle fibers [7]. Thus, the higher lipid content in the muscles of female largemouth bass may account for the larger gaps between collagen fibers. Lipids tend to accumulate in collagenous connective tissue. Excessive lipid deposition within the connective tissue leads to a more flaccid meat texture [38]. Additionally, variations in the lipid content of the muscular membrane can affect muscular membrane thickness, resulting in lower muscle fiber density and, subsequently, reduced muscle tightness [39]. Thus, the higher lipid content in female muscles may lead to a more flaccid arrangement of muscle fibers and a softer meat texture.

The muscles’ pH and centrifugal water loss are crucial sensory indicators of meat quality [40]. However, we detected no significant differences in these parameters between male and female largemouth bass. The shear force is a key indicator of muscle tenderness and is lower when muscles are more relaxed. The collagen fiber content affects the shear force [41]. The collagen fiber content was significantly higher in the muscles of male fish than in the muscles of female fish, potentially contributing to the higher shear force of male fish muscles. This suggests that the muscles (fillet) of male fish will be chewier in texture. We detected a positive correlation between the collagen fiber content in male fish muscles and Met and Arg contents, suggesting that these amino acids may positively affect collagen fiber synthesis. In further research, we intend to investigate the involvement of these two amino acids in collagen fiber synthesis in largemouth bass.

The lipid content in muscles is a significant factor influencing meat coloration. A previous study found that the L* and b* values increased with increasing muscle lipid content, and they were used as indicative parameters for spoilage in raw fillets [9]. In this study, we detected a higher lipid content in the muscles of female fish than in the muscles of male fish, which is intuitively manifested by the higher W* value. This may account for the significantly elevated color index values, including L*, a*, and b*, in the muscle tissue of female fish. The higher L* value of the female muscles may indicate increased susceptibility to oxidation [42], which would accelerate muscle deterioration and shorten its shelf life. Aside from lipids, the amino acid Tyr also contributes to variations in protein color, playing a pivotal role in the melanogenesis pathway and significantly enhancing muscle color intensity [43]. The higher Tyr content in the muscles of female fish may have contributed to the higher L*, a*, b*, and W* values. Further research is required to investigate the impact of various compounds on muscle color indices.

In summary, the male fish muscles exhibited higher shear force, indicative of a chewier meat texture. The female fish muscles had higher color index values, potentially indicative of increased susceptibility to oxidation.

### 4.3. Enhanced Apoptosis and Immunity in Male Fish Muscles

The regulation of cell proliferation, cell differentiation, and cell apoptosis is significantly influenced by various pathways, including cellular senescence, ErbB signaling, apoptosis, and ferroptosis pathways [44,45]. The processes of cell proliferation and apoptosis participate in the programmed elimination of aberrant cells, thereby contributing to the maintenance of overall health, a process that is an important part of the immune response [46,47]. We detected significant enrichment of these pathways in the muscles of male fish, indicative of a heightened immune function primarily characterized by an accelerated rate of cell clearance. Consistent with this, genes related to cellular proliferation and apoptosis, including *akt2*, *src*, and *kras*, were upregulated in male muscles. *akt2* is involved in insulin and glucose metabolism and has been shown to play a pivotal role in the inhibition of muscle growth of zebrafish and triploid crucian carp [48,49,50]. Additionally, *akt2* is involved in suppressing inflammation and enhancing resistance against pathogens [51]. In the present study, we detected upregulation of *akt2* in male fish muscles. This may have impeded muscle growth in males and/or may have interacted with numerous upstream and downstream genes to augment non-specific immunity in male fish. Previous studies have demonstrated the pivotal role of SRC in pathogen recognition and immune defense, as well as its ability to enhance phagocytosis to eliminate pathogens [52,53]. The elevated expression of this gene in male fish muscles further signifies a heightened immune function in males. In the event of cellular damage, *kras* triggers the autophagy mechanism, thereby expediting the removal of impaired organelles to maintain cellular health [54]. We detected upregulation of *kras* in male fish muscles, suggesting that the intrinsic self-purification capacity of muscle cells is elevated, thereby promoting cellular homeostasis and maintaining overall physiological well-being. Together, these results clearly demonstrate that male fish have a more robust immune regulatory mechanism than female fish in the same environment.

We detected significant enrichment of cardiac muscle contraction, adrenergic signaling in cardiomyocytes, and other pathways in the muscles of female largemouth bass. The enrichment of these pathways is indicative of the enhanced muscle contraction ability of female fish. Various studies have demonstrated that the augmentation of muscle contractile capacity and an increase in the frequency of muscle contraction activity can stimulate muscle growth, thereby contributing to an increase in fish body weight [55]. These findings imply that the enhanced growth performance of female fish may be attributed to their heightened muscle contraction capability. Our analyses identified *acta1b*, *myl13*, *myl2a*, and *tpm2* as pivotal regulators of muscle movement in female fish. Further research is required to elucidate their underlying mechanisms.

## 5. Conclusions

Under the conditions in this study, the weight gain rate of female largemouth bass was significantly higher than that of males, and this may have been because of the elevated lipid content in the muscles of females. The male fish exhibited distinctive advantages in terms of their amino acid and fatty acid profiles. Specifically, the muscles of male fish had higher contents of Ser, Val, Met, Arg, C24:1, and C18:3n3. Additionally, the collagen fiber content in the muscles was higher in male fish than in females, resulting in a higher shear force, ultimately contributing to enhanced chewiness and superior taste. The muscles of female fish were darker than those of male fish, suggesting that they are more susceptible to oxidation. Finally, although females showed a slight growth performance advantage over males, the muscles of male fish had a more robust immune regulatory mechanism, thereby effectively maintaining cellular homeostasis.

## Figures and Tables

**Table 1:** Growth parameters of *M. salmoides* and proximate composition of the muscles of male and female fishes.

	Items	Female	Male	Significance
Growth ability	Weight gain rate (%)	678.77 ± 27.51	588.15 ± 18.18	**
	feed efficiency	1.35 ± 0.03	1.26 ± 0.06	ns
	Fillet yield (%)	54.29 ± 1.12	51.35 ± 0.65	*
Proximate composition	Moisture (g/100 g)	74.9 ± 0.39	75.48 ± 0.46	ns
	Ash (g/100 g)	1.45 ± 0.06	1.53 ± 0.06	ns
	Lipid (g/100 g)	2.08 ± 0.05	1.58 ± 0.09	**
	Protein (g/100 g)	20.35 ± 0.39	20.23 ± 0.51	ns

Note: Significance is indicated by *p* < 0.05 (*) and *p* < 0.01 (**); ns, not significant (*p* ≥ 0.05).

**Table 2:** Amino acid composition of the muscles of male and female *Micropterus salmoides* (g/100 g dry matter).

	Female	Male	Significance
Asp	1.66 ± 0.03	1.57 ± 0.01	*
Thr	0.71 ± 0.01	0.72 ± 0.03	ns
Ser	0.55 ± 0.01	0.60 ± 0.01	**
Glu	2.2 ± 0.08	2.22 ± 0.05	ns
Gly	0.76 ± 0.01	0.73 ± 0.01	*
Ala	0.97 ± 0.03	0.97 ± 0.03	ns
Cys	0.17 ± 0.00	0.15 ± 0.00	*
Val	0.87 ± 0.01	0.75 ± 0.02	**
Met	0.37 ± 0.04	0.41 ± 0.00	**
Ile	0.76 ± 0.03	0.77 ± 0.03	ns
Leu	1.36 ± 0.01	1.27 ± 0.01	**
Tyr	0.49 ± 0.02	0.48 ± 0.02	ns
Phe	0.70 ± 0.02	0.69 ± 0.02	ns
Lys	1.53 ± 0.03	1.51 ± 0.04	ns
His	0.40 ± 0.01	0.42 ± 0.02	ns
Arg	0.84 ± 0.01	0.96 ± 0.03	**
Pro	0.51 ± 0.02	0.51 ± 0.03	ns
EAA	6.30 ± 0.02	6.09 ± 0.01	*
NEAA	8.58 ± 0.09	8.61 ± 0.17	ns
DAA	5.62 ± 0.07	5.49 ± 0.08	ns
TAA	14.87 ± 0.11	14.70 ± 0.18	ns
EAA/TAA	42.33 ± 0.20	41.44 ± 0.42	ns

Note: Significance is indicated by *p* < 0.05 (*) and *p* < 0.01 (**); ns, not significant (*p* ≥ 0.05). EAA, essential amino acids; NEAA, non-essential amino acids; DAA, delicious amino acids (Asp, Glu, Gly, and Ala); TAA, total amino acids.

**Table 3:** Fatty acid composition of muscles of male and female *Micropterus salmoides* (% of total fatty acids, dry matter).

	Female	Male	Significance
C14:0	1.55 ± 0.01	1.61 ± 0.07	ns
C16:0	22.42 ± 0.10	21.45 ± 0.36	*
C18:0	5.86 ± 0.07	5.79 ± 0.24	ns
SFA	28.84 ± 0.47	29.83 ± 0.16	ns
C16:1	3.82 ± 0.01	3.84 ± 0.13	ns
C18:1n-9c	30.69 ± 0.15	30.64 ± 1.05	ns
C20:1	1.17 ± 0.01	1.20 ± 0.04	ns
C22:1n-9	0.30 ± 0.02	0.38 ± 0.15	ns
C24:1	0.43 ± 0.01	0.52 ± 0.03	*
MUFA	34.57 ± 0.74	33.77 ± 0.30	ns
C20:2	0.78 ± 0.00	0.79 ± 0.03	ns
C18:2n-6	20.93 ± 0.34	21.56 ± 0.70	ns
C18:3n-3	1.73 ± 0.03	1.96 ± 0.07	*
C20:3n-6	0.23 ± 0.00	0.00 ± 0.00	**
C20:3n-3	0.22 ± 0.00	0.00 ± 0.00	**
C20:4n-6	0.80 ± 0.01	0.84 ± 0.11	ns
C20:5n-3 (EPA)	0.93 ± 0.01	1.06 ± 0.07	ns
C22:6n-3 (DHA)	8.16 ± 0.09	8.36 ± 0.68	ns
PUFA	34.57 ± 0.74	33.77 ± 0.30	ns
PUFA n-3	11.37 ± 0.67	11.03 ± 0.04	ns
PUFA n-6	22.40 ± 0.65	21.95 ± 0.34	ns
∑n-6/∑n-3	1.99 ± 0.15	1.99 ± 0.04	ns

Note: Significance is indicated by *p* < 0.05 (*) and *p* < 0.01 (**); ns, not significant (*p* ≥ 0.05). DHA, docosahexaenoic acid; EPA, eicosapentaenoic acid; SFA, saturated fatty acids; MUFA, monounsaturated fatty acids; PUFA, polyunsaturated fatty acids; PUFA n-3, omega 3 polyunsaturated fatty acids; PUFA n-6, omega 6 polyunsaturated fatty acids.

**Table 4:** Basic meat quality parameters of male and female *Micropterus salmoides*.

Items		Female	Male	Significance
Shear force (g)		65.03 ± 8.52	91.03 ± 4.74	**
Centrifugal water loss (%)		22.63 ± 1.14	22.7 ± 0.84	ns
pH		6.18 ± 0.03	6.25 ± 0.05	ns
Color	a*	−0.72 ± 0.28	0.15 ± 0.21	*
	b*	−2.11 ± 0.22	−0.41 ± 0.11	**
	L*	38.39 ± 1.81	31.64 ± 0.99	*
	W*	38.35 ± 1.81	31.63 ± 0.99	*

Note: Significance is indicated by *p* < 0.05 (*) and *p* < 0.01 (**); ns, not significant (*p* ≥ 0.05).

## Data Availability

Data will be made available on request.

## Supplementary Materials

The following supporting information can be downloaded at https://www.mdpi.com/article/10.3390/biology13121029/s1. Table S1: Primers used for qRT-PCR in this study; Table S2: Transcriptome sequencing results for male and female *Micropterus salmoides*; Table S3: KEGG pathway analysis of the muscles of *Micropterus salmoides*. Table S4: Correlation analysis between genes and muscle content of amino acids and fatty acids in females and males.
