# Supplementary material for: Characteristic Muscle Quality Parameters of Male Largemouth Bass (Micropterus salmoides) Distinguished from Female and Physiological Variations Revealed by Transcriptome Profiling
Source: Biology (Basel). 2024 Dec 8;13(12):1029. doi: 10.3390/biology13121029 (PMC11672892; doi:10.3390/biology13121029)
Supplement: Supplementary file 1 [file biology-13-01029-s001.zip › biology-3338615-supplementary.pdf]

Supplementary material:

Table S1 Primers used for qRT-PCR in this study

Gene	Forward (5'--3')	Reverse (5'--3')
β-actin	ATCGCCGCACTGGTTGTTGAC	CCTGTTGGCTTTGGGGTTC
lpl	GAGCAGCCGATGAAGATT	CAGTGGTGATGAGGAAGG
pla2g3	TCTGATGGAGGCGAATGA	TGAATGTGGAGGTAGTAACG
tyrp1b	CTGTGAGAGTGTGGATGAC	AGGAGGTGGAGTAATAAGGT
LOC119886780	GCTCTTCTGTATGCTCTGTA	CCTTCAACTAACTGGACCTT
LOC119896779	CTGATGGAACTGTGAAGAGA	TTGAAGGCTGTTGGCATT
scdb	TCCTTGATTGTGGCATACTT	GGCGGTGGTGATATTGAG
dgat2	TCCTTGATTGTGGCATACTT	GGCGGTGGTGATATTGAG
bhmt	GCAAGAGTGAGGATGATGT	AGTCAGGAGTGTGGTAGG
tecrb	CGAGGTGGAGATACTGGAT	AGGATGGCTCTTGTGGAA
LOC119910000	CAAGGCTGACAGTGACAA	CGATAGTGACATCTGAGGTT
LOC119909999	GAATGGAGACAAGGCGATA	TCATATACACTGCGGACTG
hsd3b7	AATGGAATCGGCACAGAG	ACAGAGTAGATGATGGTATCG

Table S2 Transcriptome sequencing results for male and female *Micropterus salmoides*

Sample	Raw Data	Clean Data	Valid Ratio	Q20%	Q30%	GC content%
Female_1	42413476	41295356	97.36	99.97	96.90	49.50
Female_2	42697362	40934514	95.87	99.97	96.48	50.50
Female_3	42542364	41278282	97.03	99.97	96.67	50
Female_4	42319828	40774216	96.35	99.97	96.69	50.50
Male_1	42723854	41013120	96.00	99.98	96.95	49.50
Male_2	42306324	40125512	94.85	99.97	96.56	50.50
Male_3	42693582	40694400	95.32	99.97	96.61	50.50
Male_4	43221454	42065524	97.33	99.97	96.50	50

Table S3 KEGG pathway on muscle of *Micropterus salmoides*

Pathway ID	KEGG Level 2	Pathway Name	P
msam04260	Organismal Systems	Cardiac muscle contraction	0.00
msam04261	Organismal Systems	Adrenergic signaling in cardiomyocytes	0.00
msam04020	Environmental Information Processing	Calcium signaling pathway	0.00
msam04310	Environmental Information Processing	Wnt signaling pathway	0.00
msam04145	Cellular Processes	Phagosome	0.00
msam04218	Cellular Processes	Cellular senescence	0.00
msam04080	Environmental Information Processing	Neuroactive ligand-receptor interaction	0.00
msam04371	Environmental Information Processing	Apelin signaling pathway	0.00

msam04810	Cellular Processes	Regulation of actin cytoskeleton	0.00
msam04916	Organismal Systems	Melanogenesis	0.00
msam04510	Cellular Processes	Focal adhesion	0.00
msam04512	Environmental Information Processing	ECM-receptor interaction	0.00
msam04530	Cellular Processes	Tight junction	0.00
msam00140	Metabolism	Steroid hormone biosynthesis	0.00
msam04350	Environmental Information Processing	TGF-beta signaling pathway	0.00
msam00830	Metabolism	Retinol metabolism	0.00
msam04270	Organismal Systems	Vascular smooth muscle contraction	0.00
msam04920	Organismal Systems	Adipocytokine signaling pathway	0.00
msam04060	Environmental Information Processing	Cytokine-cytokine receptor interaction	0.00
msam04012	Environmental Information Processing	ErbB signaling pathway	0.00
msam00630	Metabolism	Glyoxylate and dicarboxylate metabolism	0.00
msam04330	Environmental Information Processing	Notch signaling pathway	0.00
msam04210	Cellular Processes	Apoptosis	0.00
msam04660	Organismal Systems	T cell receptor signaling pathway	0.00
msam04010	Environmental Information Processing	MAPK signaling pathway	0.01
msam04217	Cellular Processes	Necroptosis	0.01
msam04514	Environmental Information Processing	Cell adhesion molecules	0.01
msam04380	Organismal Systems	Osteoclast differentiation	0.01
msam05410	Human Diseases	Hypertrophic cardiomyopathy	0.01
msam04140	Cellular Processes	Autophagy - animal	0.01
msam04115	Cellular Processes	p53 signaling pathway	0.01
msam01040	Metabolism	Biosynthesis of unsaturated fatty acids	0.01
msam04110	Cellular Processes	Cell cycle	0.01
msam04150	Environmental Information Processing	mTOR signaling pathway	0.01
msam04621	Organismal Systems	NOD-like receptor signaling pathway	0.01
msam03015	Genetic Information Processing	mRNA surveillance pathway	0.01
msam04672	Organismal Systems	Intestinal immune network for IgA production	0.01
msam04064	Environmental Information Processing	NF-kappa B signaling pathway	0.02
msam00561	Metabolism	Glycerolipid metabolism	0.02
msam04610	Organismal Systems	Complement and coagulation cascades	0.02
msam04662	Organismal Systems	B cell receptor signaling pathway	0.02
msam00010	Metabolism	Glycolysis / Gluconeogenesis	0.02
msam04370	Environmental Information Processing	VEGF signaling pathway	0.02

msam05414	Human Diseases	Dilated cardiomyopathy	0.02
msam04933	Human Diseases	AGE-RAGE signaling pathway in diabetic complications	0.02
msam04360	Organismal Systems	Axon guidance	0.02
msam00120	Metabolism	Primary bile acid biosynthesis	0.03
msam05168	Human Diseases	Herpes simplex virus 1 infection	0.03
msam04668	Environmental Information Processing	TNF signaling pathway	0.03
msam00981	Metabolism	Insect hormone biosynthesis	0.03
msam04910	Organismal Systems	Insulin signaling pathway	0.03
msam00620	Metabolism	Pyruvate metabolism	0.03
msam00565	Metabolism	Ether lipid metabolism	0.03
msam00533	Metabolism	Glycosaminoglycan biosynthesis - keratan sulfate	0.03
msam00900	Metabolism	Terpenoid backbone biosynthesis	0.03
msam00591	Metabolism	Linoleic acid metabolism	0.03
msam00592	Metabolism	alpha-Linolenic acid metabolism	0.03
msam00590	Metabolism	Arachidonic acid metabolism	0.03
msam03320	Organismal Systems	PPAR signaling pathway	0.03
msam04914	Organismal Systems	Progesterone-mediated oocyte maturation	0.03
msam00520	Metabolism	Amino sugar and nucleotide sugar metabolism	0.04
msam04976	Organismal Systems	Bile secretion	0.04
msam04620	Organismal Systems	Toll-like receptor signaling pathway	0.04
msam04630	Environmental Information Processing	JAK-STAT signaling pathway	0.04
msam04142	Cellular Processes	Lysosome	0.04
msam04068	Environmental Information Processing	FoxO signaling pathway	0.04
msam00350	Metabolism	Tyrosine metabolism	0.04
msam00515	Metabolism	Mannose type O-glycan biosynthesis	0.04
msam00500	Metabolism	Starch and sucrose metabolism	0.05
msam00062	Metabolism	Fatty acid elongation	0.05
msam00640	Metabolism	Propanoate metabolism	0.05
msam05202	Human Diseases	Transcriptional misregulation in cancer	0.05
msam00260	Metabolism	Glycine, serine and threonine metabolism	0.05
msam00860	Metabolism	Porphyrin metabolism	0.05

Table S4 Correlation analysis between genes and muscle content of amino acids and fatty acids in female and male

genes	items	correlation index r	P	correlativity
bhmt	Ser	0.86494743	0.00138889	Positive
bhmt	Val	0.89612792	0.00138889	Positive
bhmt	Met	0.86877987	0.02222222	Positive
bhmt	Arg	0.56209315	0.07083333	Positive
bhmt	C24:1	0.6782659	0.00138889	Positive
bhmt	C18:3n3	0.70713399	0.02777778	Positive
bhmt	cf	0.62090718	0.09444444	Positive
tecrb	Ser	0.79485497	0.07361111	Positive
tecrb	Val	0.84641271	0.06805556	Positive
tecrb	Met	0.85300198	0.04444444	Positive
tecrb	Arg	0.60198412	0.0375	Positive
tecrb	C24:1	0.57964975	0.075	Positive
tecrb	C18:3n3	0.66063657	0.06944444	Positive
tecrb	cf	0.67021803	0.03888889	Positive
hsd3b7	Ser	0.83056338	0.03611111	Positive
hsd3b7	Val	0.87104817	0.03333333	Positive
hsd3b7	Met	0.82371463	0.08888889	Positive
hsd3b7	Arg	0.58084473	0.04444444	Positive
hsd3b7	C24:1	0.58218232	0.07361111	Positive
hsd3b7	C18:3n3	0.64593696	0.08194444	Positive
hsd3b7	cf	0.6349067	0.05972222	Positive
lpl	Asp	0.59277059	0.01944444	Positive
lpl	Gly	0.51669621	0.03055556	Positive
lpl	Cys	0.50572361	0.09722222	Positive
lpl	Leu	0.95938765	0.01111111	Positive
lpl	C16:0	0.48737663	0.06944444	Positive
lpl	C20:3n6	0.97509786	0.05833333	Positive
lpl	C20:3n3	0.97971075	0.025	Positive
scdb	Asp	0.54851882	0.04861111	Positive
scdb	Gly	0.49619252	0.03611111	Positive
scdb	Cys	0.67044057	0.00833333	Positive
scdb	Leu	0.91771015	0.06666667	Positive
scdb	C16:0	0.53043005	0.05833333	Positive
scdb	C20:3n6	0.99664224	0.00833333	Positive
scdb	C20:3n3	0.99249069	0.09166667	Positive
dgat2	Asp	0.53965625	0.04166667	Positive
dgat2	Gly	0.46989884	0.075	Positive
dgat2	Cys	0.60955399	0.1	Positive
dgat2	Leu	0.92957073	0.05555556	Positive
dgat2	C16:0	0.55376412	0.03888889	Positive
dgat2	C20:3n6	0.99802089	0.08333333	Positive

dgat2	C20:3n3	0.99843905	0.03333333	Positive
